# Modeling the residential distribution of enrolled students to assess boundary-induced disparities in public school access

**DOI:** 10.1371/journal.pone.0222766

**Published:** 2019-10-01

**Authors:** Louie John M. Rubio, Damian N. Dailisan, Maria Jeriesa P. Osorio, Clarissa C. David, May T. Lim

**Affiliations:** 1 National Institute of Physics, University of the Philippines Diliman, Quezon City, Philippines; 2 College of Mass Communication, University of the Philippines Diliman, Quezon City, Philippines; Johns Hopkins University, UNITED STATES

## Abstract

Given school enrollments but in the absence of a student residence census, we present a gravity-like model to infer the residential distribution of enrolled students across various administrative units. Multi-scale analysis of the effects of aggregation across different administrative levels allows for the identification of administrative units with sub-optimally located schools and highlights the challenges in allocating resources. Using this method, we verify that the current scheme of free cross-enrollment across administrative boundaries is needed in achieving universal education in the Philippines.

## Introduction

Achieving inclusive and quality education is part of the 2030 Agenda adopted at the United Nations Sustainable Summit in 2015 [1]. As with most Sustainable Development Goals (SDGs) targets, it uses index indicators [2, 3], such as the net enrollment rate (NER) or the fraction of enrolled students in a specific age group, to track progress. A downside of the NER is that it is prone to misestimation because of its dependence on population projections [4]. Furthermore, the averaging process masks factors that affect late school-entry such as poverty [5], perceptions on school readiness [5], and distance of schools [4].

Information-based initiatives [6] to reform the public school system in the Philippines range from an online volunteer-driven participatory monitoring school-level platform [7] to data collection using high-resolution surveys required by the Department of Education (DepEd) either at the school-level (EBEIS) or at the learner-level (LIS) [8]. The data collection process itself is usually quite involved and has to meet the challenges of data standardization and timely collection [6].

Accessibility models that incorporate spatial detail can range from the simple gravity-based model [9], similar to the one we use in this paper, to highly detailed models that include topography, land use plans [10], and navigation tools. To date, geospatial studies of Philippine schools have focused on understanding location-influenced factors. Linked to natural disasters and civil unrest are findings that: (a) the repeated use of school structures as evacuation centers has a negative impact on school performance [11]; and (b) that at a provincial-level (Admin Level 2) aggregation, schools with poor facilities (number of classrooms, toilets, and seats) geographically correlates to areas exposed to frequent natural calamities and civil unrest [12].

Premature aggregation by administrative levels, which is not accounted for when dealing with raw population figures, may skew key indicators. Recommendations based on analysis done at the provincial level (Admin Level 2) [12] might miss out on the immediate needs of schools at the municipal (Admin Level 3) or even at the barangay level (Admin Level 4).

In education system planning, the target is to build enough schools so that all school-aged children can attend and complete primary and secondary-level education. For primary schools in the Philippines, a DepEd order outlines the priorities of the government: (a) completion of incomplete public elementary schools (i.e. schools that do not offer all grade levels); and (b) building at least one public elementary school per barangay in the country [13]. Though land acquisition is an important consideration in selecting school locations, DepEd no longer uses its own funds to purchase school sites or properties since 1994. Instead, DepEd relies on land donations from local governments or private entities [14] under the Contract of Usufruct, which provides the “full use of the property for free without any payment for as long as the property is used for educational purposes” [13]. Thus, scenarios where schools are constructed in non-ideal locations can occur.

Public schools in the Philippines accept students regardless of place of residence. Though physical proximity is expected to influence school attendance or enrollment, it is still the prerogative of parents to enroll their children in their preferred (e.g. private, specialized, or religious) schools. Nonetheless, poor physical accessibility of schools likely results in systemic disadvantages for poorer households. For one, longer commutes are more expensive and time-consuming. Surprisingly, poorer households do not necessarily live further away from schools. In urban settings, locations of urban poor settlements are linked to the accessibility of public transportation [15].

In the United States, Figlio *et al*. [16] found a significant correlation between real-estate prices and school performance. However, Albacea *et al*. [17] determined that the density of poor households in barangays are not associated with the presence or absence of elementary schools, churches, and plazas. Rather, real-estate prices in the Philippines are influenced by the increase in master-planned townships and the construction of shopping malls [18]. School quality does not factor greatly in school choice compared to the US since parents have limited access to information on school performance and only have reputational information about school quality from inter-school contests [6].

School overcrowding due to increasing student enrollment and continuing budget constraints have led to different crowding alleviation policies. In the US, multi-track year-round calendars are implemented in some public schools as a potential solution to overcrowding [19]: the student body is split into different tracks with staggered schedules (with some students attending while others are on break) to increase enrollment per facility. In the Philippines, a double shift policy is implemented in some areas to alleviate classroom shortage [20]. Double shift sessions for classes split students into morning and afternoon shifts [21].

Due to the general belief of parents that children will get a better education in uncrowded classrooms, school capacity was identified as a determining factor for school attendance [22]. Except for multi-shift classes, the pupil-teacher ratio (PTR) acts as a proxy for class size. Though schools implementing multiple shifts are not explicitly tagged in the DepEd dataset, we infer that schools with high PTRs (beyond 200) is likely due to the adoption of double shift sessions. The Philippines has a higher median PTR for 2013 (37.7) compared to the worldwide average PTR (23.54) [23].

The teaching-learning process is also influenced by the quality of teachers themselves [22]. While teachers in the Philippines have the freedom to choose which schools they want to teach in, the availability of budget items for teaching positions constrains where they can apply [24].

In this work, we use the geolocation of schools along with enrollment data to create a reachability map that shows the effective number of elementary schools from the perspective of primary school students. We then develop an index to assess the accessibility of elementary schools, specifically the accessibility of elementary schools to the centroids of the most local administrative unit (MLAU). Including the MLAU population data, we assess the effectivity of the public school system and gauge the impact of the spatial distribution of population on the school system. To demonstrate, we use enrollment data from the Philippines and the United Kingdom.

## Methods

To quantify the accessibility of elementary schools to barangays, the following datasets were used: 1) location data for schools in the Philippines, 2) shapefile data for the Philippines [25], and 3) the September 2017 Philippine Standard Geographic Code (PSGC) publication [26]. Datasets for the United Kingdom primary school system were also used for comparison [27–29].

### Datasets

The DepEd dataset includes school coordinates, population broken down by year level from 2012-2016 for all public schools, and school IDs. Barangay-level shape and population data for 2002 from the Philippine Statistics Authority were also used for visualization and data cleaning. The 2017 PSGC dataset, which contains identifiers for geographic areas in the Philippines at different scales, was used to update the provincial-level geographic codes in the 2002 shapefiles. To clean the school coordinates, a bounding box encompassing the Philippines was used to remove outlying points. Schools without location data, erroneous coordinates, or misassigned DepEd regions were removed. A total of 36,461 public elementary schools were used in this analysis.

For the primary school system in England, a dataset containing names and coordinates of schools [28] was merged with school capacity data for the academic year 2016-2017 from the Department for Education of the United Kingdom [27]. In total, we considered 16,520 primary schools for the 326 districts in England.

### School influence

The influence of a school is treated as a function that varies with distance *r* from the school. Students in locations within a distance *r* < *r*_*c*_ from a school considers that school as an option. Students may still choose schools that are at a distance *r* > *r*_*c*_ from their residence, but with a lower likelihood than other schools that are closer to the students’ location.

Mathematically, we express this as a clipped power law given by

$$\phi \left(r\right)=\text{min}\{1,{\left(\frac{r}{r_{c}}\right)}^{-\alpha }\},$$ (1)

where *r*_*c*_ is a reasonable distance from a school, and *α* dictates how fast the effective range decays. This formulation of the potential is similar to the gravity model [9]. For example, choosing *r*_*c*_ = 5 km assigns *ϕ* = 1 for any location within a five-kilometer radius from a school. Locations beyond *r*_*c*_ experience a potential that varies according to the power law expression given in Eq 1, i.e. the said school is a less likely choice than a school at a closer distance. For this work, we arbitrarily set *α* = 2 and *r*_*c*_ = 5 km.

From the definition of the potential, we can infer the distance of the nearest school to a barangay based on the potential experienced by the barangay. For a given potential value *ϕ*, the closest school can be expressed as $r=r_{c}\phi^{-\frac{1}{\alpha }}$. For *ϕ* = [10^−1^, 10^−2^, 10^−3^, 10^−4^] gives *r* ≈ [15, 50, 158, 1581] km.

### The number of accessible schools

For the number of accessible schools at different points in the entire country, one can sum the potential contributions of all schools *i* at the location of barangay *b*, ∑_*i*_ *ϕ*(*r*_*ib*_), where *r*_*ib*_ is the distance between school *i* and barangay *b*. This approximately counts the number of schools within *r* < *r*_*c*_. The accessibility is sampled at the locations of the most local administrative unit (MLAU), which in the Philippines is the barangay. Henceforth, we refer to barangays and MLAUs interchangeably. Barangay information was obtained using a modified version of the 2002 shapefile that has updated provinces from the September 2017 PSGC publication [26].

### Treatment of administrative boundary levels

All measurements are sampled at the level of the barangay, which in turn compose the municipalities/cities [30]. Aggregation can then be done on the next administrative levels (with decreasing granularity: barangay, municipality/city, provincial, and regional) by summing all the measurements from contributing MLAUs. The effect of enforcing regional (administrative) boundaries on the access to schools were also examined.

### Modeling the number of enrolled students

While we can estimate the population that is of a particular age range (and thus have an estimate of the number of residents eligible for a particular grade level), our data do not provide us with an accurate estimate of the number of actual students that are enrolled within administrative units. Instead, we will use the potential formulation and reported enrollment figures to estimate where enrolled students reside.

We make the assumption that enrolled students would reside within some radius from the school, and administrative units with a larger population should have more enrolled students. Given actual enrollment figures and geographic distributions of schools, we then calculate the number of enrolled students at the MLAU.

We assume that the school population at school *i* is distributed among barangays, such that the number of students *S*_*ib*_ from barangay *b* that attend school *i* is given by

$$S_{ib}={\left(\text{school}\;\text{population}\right)}_{i}\times \frac{\left(\text{MLAU}\;\text{population}\right)\phi_{ib}}{\sum_{b}\left(\text{MLAU}\;\text{population}\right)\phi_{ib}}.$$ (2)

The school population at *i* is proportional to the potential *ϕ*_*ib*_ at barangay *b* due to school *i*, weighted by the barangay population, relative to the potential exerted by the school on all other barangays. The number of students in a barangay *b* is ∑_*i*_ *S*_*ib*_, and the modeled number of students in an administrative unit *k* is ∑_*b*∈*k*_ ∑_*i*_ *S*_*ib*_.

### Effectivity of public school access and enrollment pressure

The potential based method described above provides an estimate of the number of enrolled students at all administrative levels. We can use our model to determine the extent to which the public school system fulfills the needs of students. From the modeled number of students, two metrics, *effectivity* and *pressure*, are proposed to evaluate the performance of the public school system.

The effectivity *E*_*k*_ of the public school system within an administrative unit *k* ∈ {barangay, municipal, provincial, regional} is defined as

$$E_{k}=\frac{\text{modeled}\;\text{number}\;\text{of}\;\text{students}\;\text{in}\;\text{an}\;\text{administrative}\;\text{unit}\;k}{\text{school}\;\text{aged}\;\text{residents}\;\text{at}\;\text{an}\;\text{administrative}\;\text{unit}\;k}.$$ (3)

The effectivity measures the fraction of children that are enrolled in an administrative unit. The number of school-aged residents in an administrative unit corresponds to the subset of the population at the grade level of interest. For Grade 4, for example, this is computed by taking the percentage of the national population that is estimated to be 10 years old for each barangay. The percentage is calculated under the assumption that there is a flat distribution in the 10-14 age range. Since the age range 10-14 corresponds to 10% of the population, then 2% of the population are 10-year-olds. The 2002 barangay population included with DATOS barangay shapefiles [25] were adjusted using the ratio of the 2015 to 2002 Philippine population, before computing the school-aged residents for each barangay.

We also define the pressure *P*_*k*_ exerted by students on the public school system as

$$P_{k}=\frac{\text{modeled}\;\text{number}\;\text{of}\;\text{students}\;\text{in}\;\text{an}\;\text{administrative}\;\text{unit}\;k}{\text{number}\;\text{of}\;\text{school}\;\text{slots}\;\text{in}\;\text{an}\;\text{administrative}\;\text{unit}\;k}.$$ (4)

Pressure measures the ratio of the demand for school slots to available slots.

For England, the number of students is similarly modeled with districts as the MLAU, with no further aggregation.

## Results and discussion

Our potential based approach takes into account the distance between barangays and schools rather than actual travel times. While travel times and distances are usually correlated, distance rather than travel time is the primary concern in choosing schools [4]. Studies on Philippine school children tracked in the Cebu Longitudinal Health and Nutrition Survey [31] found that typical travel times for children in urban areas have increased from 18 minutes in 1998 to 30 minutes in 2005. A more recent survey in 2014 estimates this at 73 minutes for the National Capital Region [32], though the survey involved all types of commuters. With a conservative estimate of 30 km/h speeds in Philippine cities, students would live 15 to 35 kilometers away from schools.

Traffic congestion policies like staggered work hours are present in Manila, e.g. schools typically begin at 7:00AM-7:30AM, offices open at 8:00AM-9:00AM, and commercial establishments open at 10:00AM. But to our knowledge, there is no systematic study that looks into how the staggered work hours scheme alleviates traffic congestion; the sheer volume of private vehicles on the road seem to be immune to volume reduction efforts. It is common for families to drop off school children using their own cars before proceeding to their workplace, which may be in a different city. Anecdotally, we know that schools (particularly private schools) contribute significantly to congestion, as traffic is noticeably lighter during class suspensions in the city.

In the context of the Philippine public school system, we assumed that all schools are similar in terms of quality. Differentiation would be relevant when private, specialized, and religious schools are considered. Such schools would lead to heterogeneity of school choice preferences, which could be accounted for by setting individual values of *r*_*c*_ for each type of school. Likewise, other factors such as the availability of transportation, duration of travel, and school quality can also affect the individual *r*_*c*_ value of a school. Schools that are more appealing to parents will have larger *r*_*c*_ values. A more realistic model would have a distribution of *r*_*c*_ values, which would be associated with the preferences of parents for *good* schools.

### Accessibility of schools

One way to describe the accessibility of education is to examine the reach (henceforth referred to as potential) of elementary schools. The relative accessibility to education for different parts of the country can be described by the school potential since it is analogous to the effective number of schools for a point on the map. Nationally, the highest value for the school potential is found in the nation’s capital and its neighboring areas. Other local maxima for the school potential are associated with metropolitan cities. Mainland Luzon has the highest values for school potential compared to other island groups in the country. A higher value of school potential suggests that there are more schools within a reasonable distance from the barangay centroid. Most barangays that have low school potential are found in island provinces such as Palawan and Tawi-Tawi. On the other hand, barangays that have large school potentials are found in the National Capital Region (NCR).

### Estimating the residential distribution of enrolled students

One way to model the residential assignment of enrolled students is to pose an assignment problem that minimizes some cost function (which may be a function of travel distance, travel time, apartment and transportation costs) subject to barangay population constraints. However, this approach might not be as realistic, as it may fail to account for other factors and interdependencies that influence where people live.

**Fig 1:**
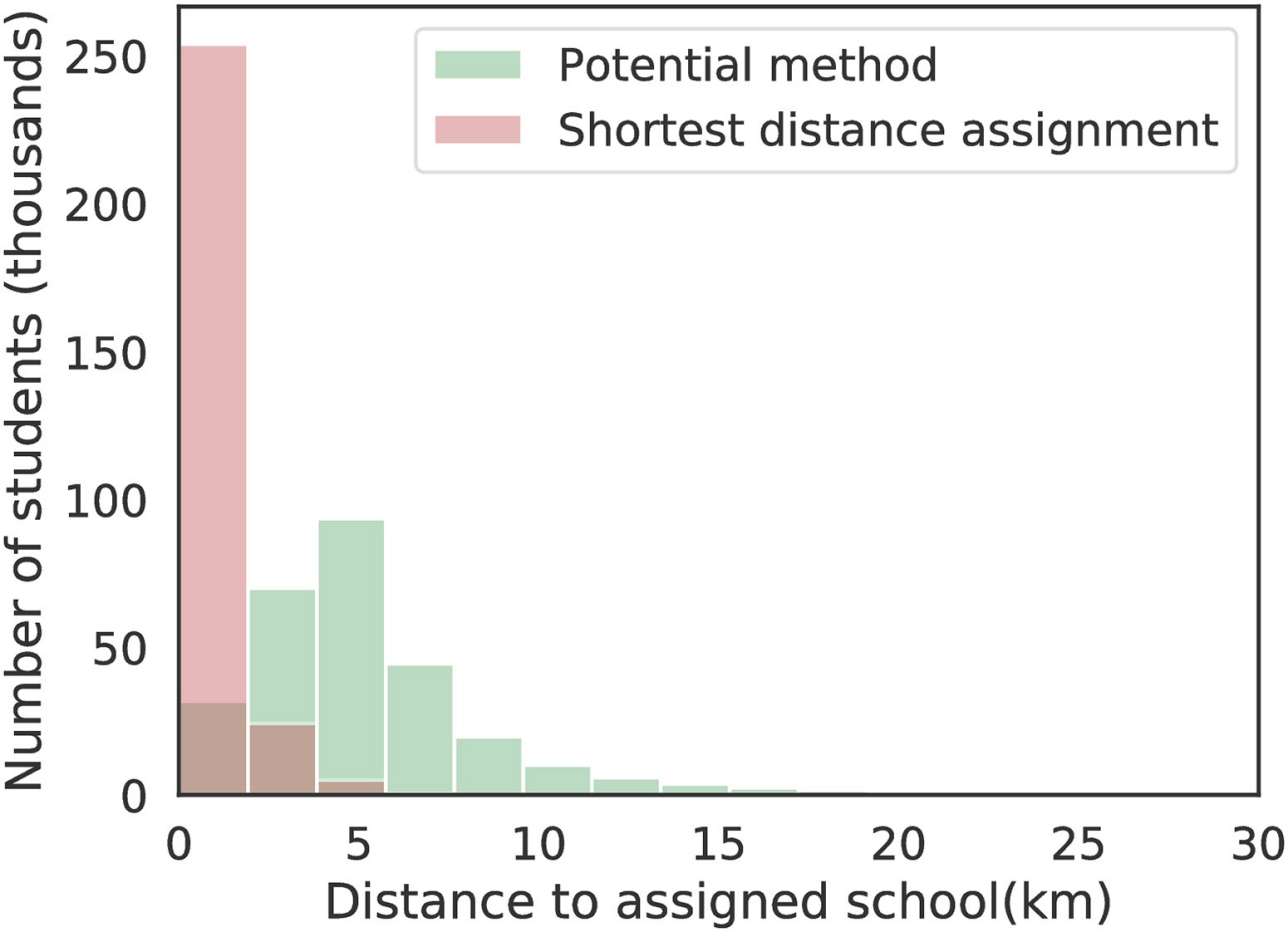
Comparison of the distributions of the modeled number of students using the shortest distance assignment with the potential model. The potential model allows for students to be situated further from schools.

Fig 1 shows a comparison between shortest distance assignment and the potential method applied to schools and barangays in NCR. The matching method greedily assigns students to the nearest school. Our potential method with *α* = 2 and *r*_*c*_ = 5 km relaxes the constraint of shortest distance assignment, allowing for students to be located further from schools. One can easily recover the maximum matching result by setting *α* to a large number, and *r*_*c*_ to a small number. We believe that our potential based approach is more robust, as the interdependence of barangays would be encoded in *S*_*ib*_.

### Pressure on the school system

**Fig 2:**
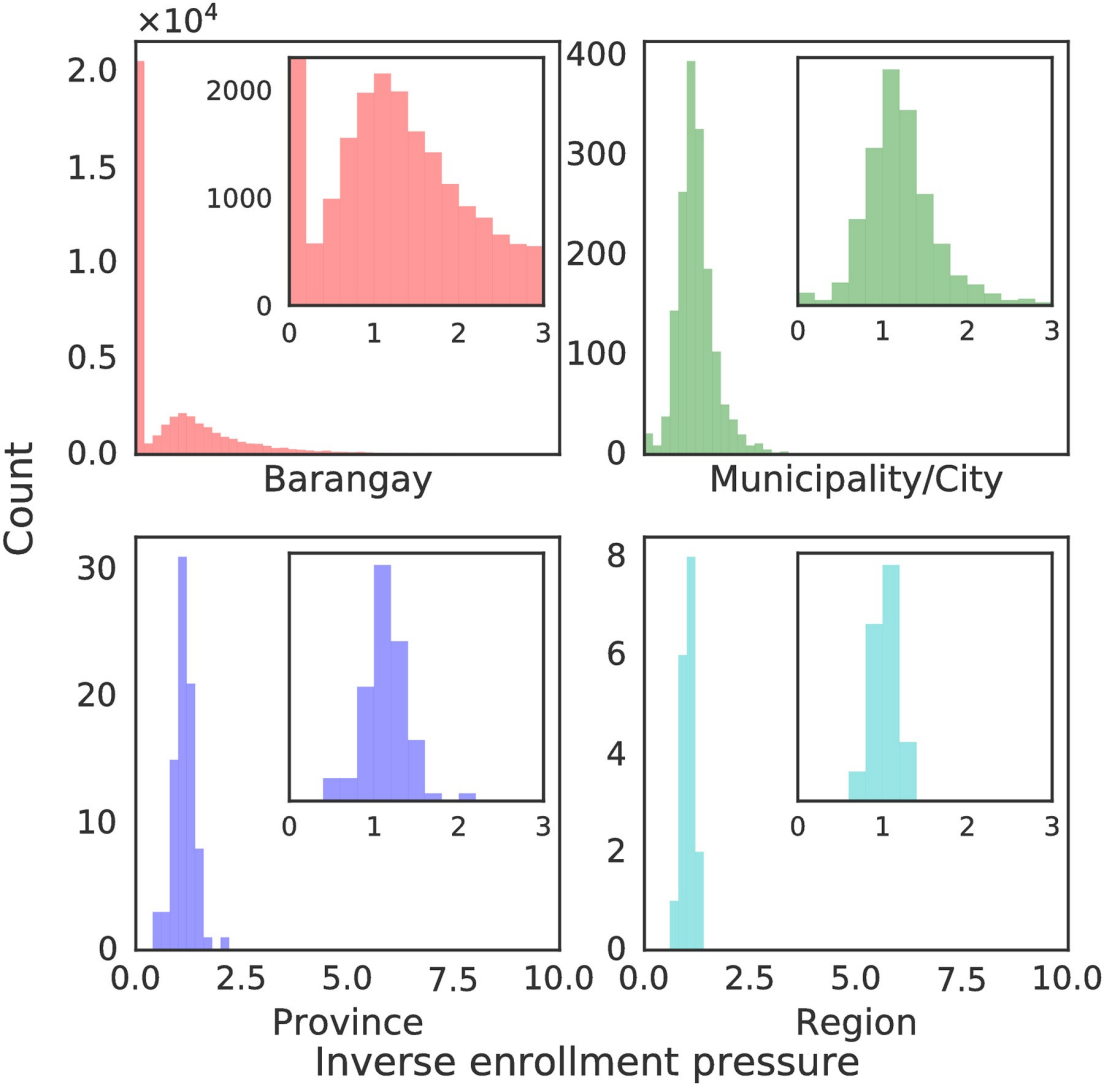
Distribution of the inverse enrollment pressure ($\frac{1}{P}$) exerted by the population on the public school system aggregated at different administrative levels.

Fig 2 shows the inverse enrollment pressure exerted by the student population on the public elementary school system aggregated at different administrative levels. The inverse enrollment pressure is used since some barangays do not have public schools, resulting in infinite values of *P*. Values of enrollment pressure *P* > 1 imply that there are more students than slots in these administrative units. For the distributions in Fig 2, this corresponds to barangays with $\frac{1}{P}<1$. Barangays with lower enrollment pressure have more slots than students within range. On the other hand, barangays with high enrollment pressure accommodate enrollees living well outside the local area (e.g. barangays hosting boarding schools). Values of $\frac{1}{P}>10$ (with a maximum value of 192) are found in 617 out of 41,940 barangays (1.47%). As the scale is coarsened from the barangay to the city/municipality, to the provincial, and eventually regional level, an expected narrowing of the spread of the distribution of inverse pressure values is observed.

**Fig 3:**
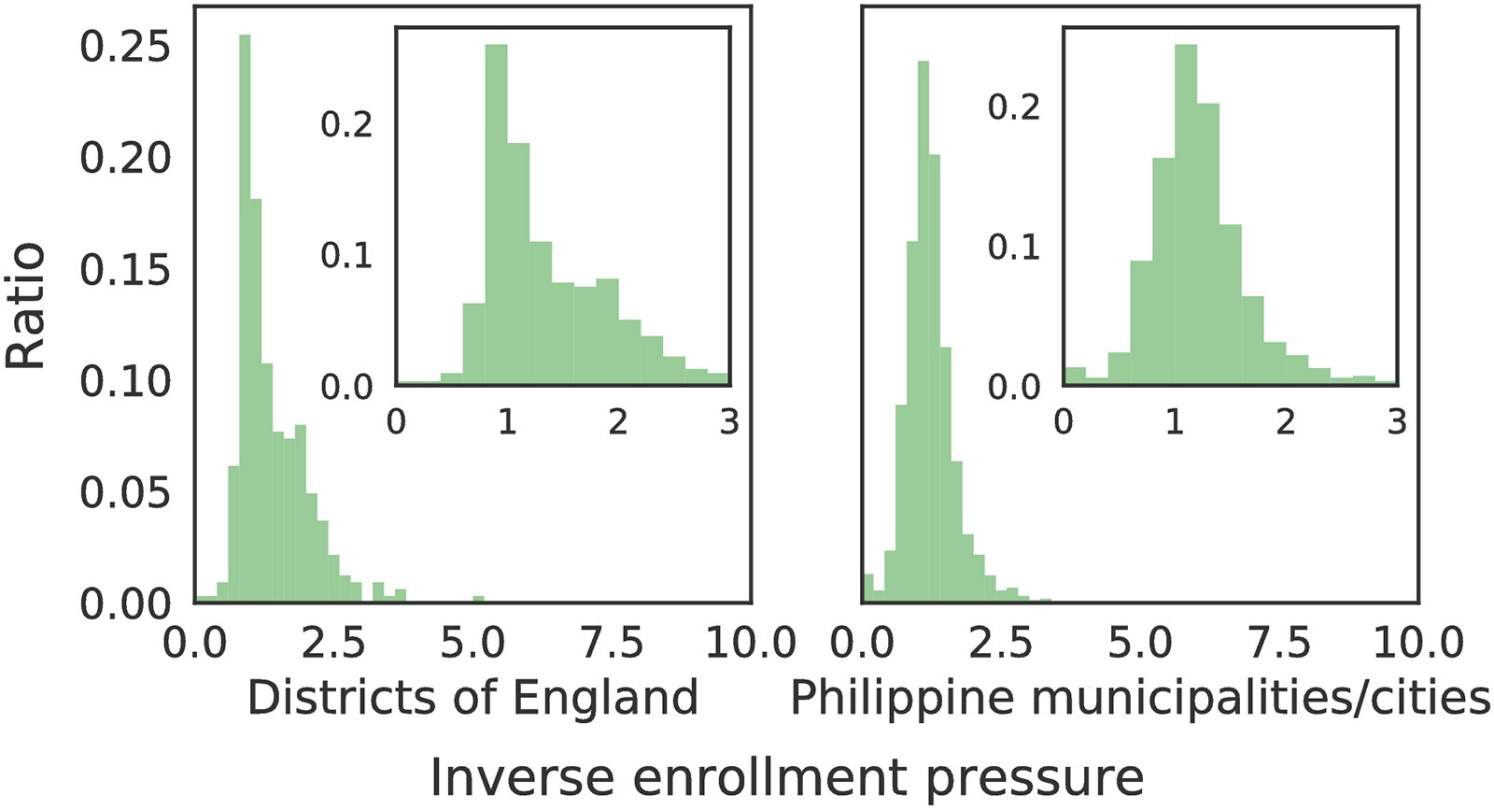
Distribution of the inverse enrollment pressure ($\frac{1}{P}$) exerted by the population on the UK public school system and Philippine public school system at the district and municipality/city level, respectively.

To benchmark the inverse pressure metric, Fig 3 shows $\frac{1}{P}$ for the primary school system in England compared to the public elementary school system of the Philippines. A district in England is administratively similar to the city/municipality in the Philippines. Since there are fewer districts in England (326) than municipalities/cities (1623) in the Philippines, the histograms were normalized for comparison. The distribution of $\frac{1}{P}$ differs largely at the extremes. Significantly more Philippine municipalities are underserved. The mode of the inverse enrollment pressure distributions is near unity, indicating generally matched values of students and available slots. The outliers measure mismatches. Roughly 0.3% (1/326) of districts in England have $\frac{1}{P}$ values less than 0.2. Schools in these districts can only accommodate 2 out of 10 students applying in the district. In comparison, around one percent (21 out of 1623) municipalities/cities in the Philippines have $\frac{1}{P}$ values less than 0.2. Areas with $\frac{1}{P}>1$ locate schools with more slots than student applicants within the potential range. England has more schools with $\frac{1}{P}>2$, which can be attributed to the presence of boarding schools.

### Effectivity of public school access

**Fig 4:**
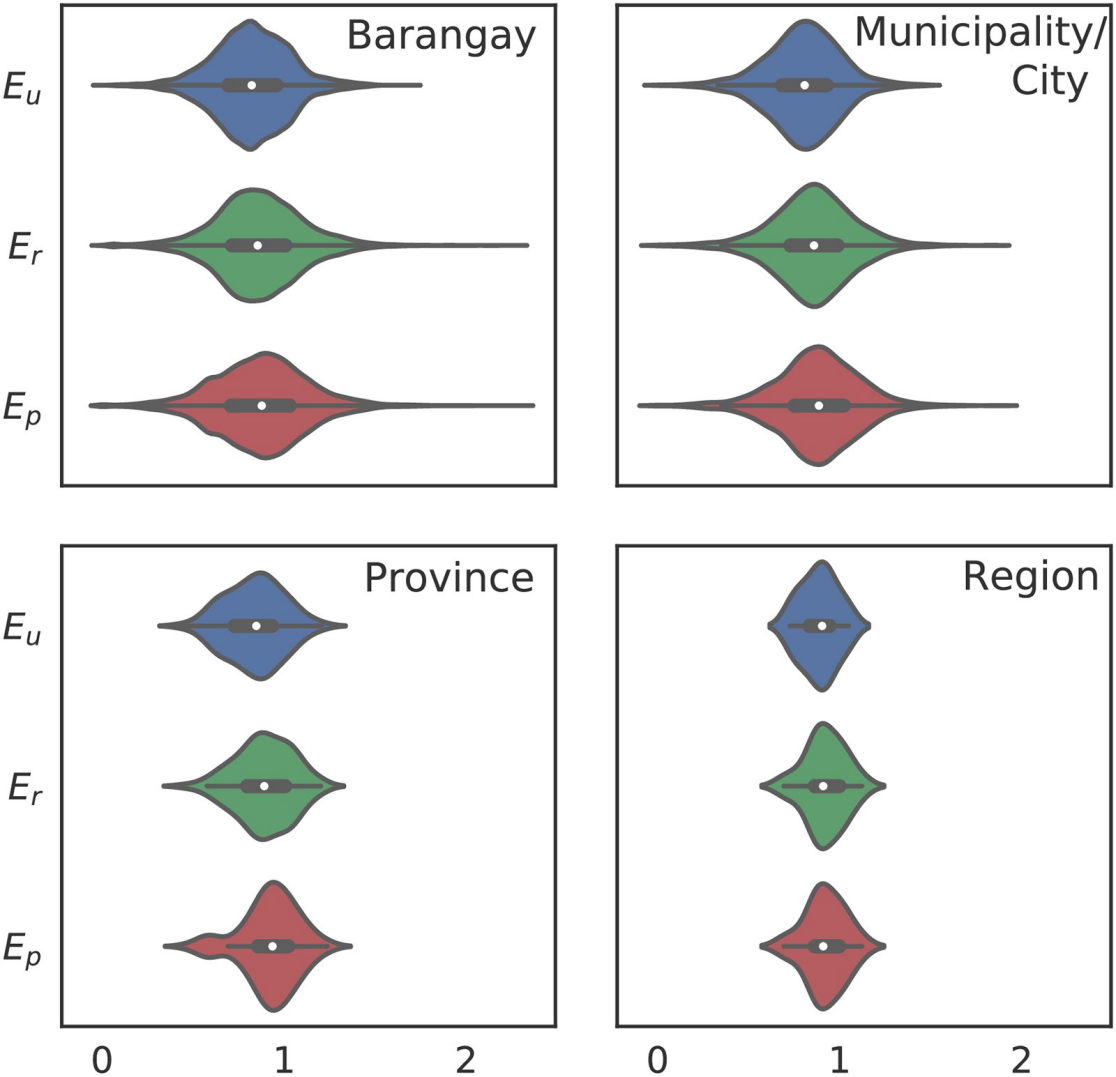
Distribution of the effectivity *E* of barangays in the Philippines for the public elementary school system at different aggregation levels without imposed boundaries (*E*_*u*_), with imposed regional boundaries (*E*_*r*_), and with imposed provincial boundaries (*E*_*p*_).

Fig 4 shows effectivity *E* for different levels of aggregation. Effectivity values *E* > 1 suggests that a particular administrative unit is accommodating students from another unit. At the barangay level, 33,995 out of 41,940 barangays have *E* < 1. Since the number of students coming from the barangay is less than the student-aged population, the barangay is unable to cater to its resident population.

The spread of the distribution decreases as the aggregation level is changed from barangays to cities/municipalities, to provincial, and finally to regional levels (Fig 4). Aggregation masks out areas that have extreme values of effectivity. The implication of Fig 4 would have been lost had we simply used NER to describe enrollment. We found effectivity values less than unity for 85% (1394/1623) of municipalities/cities, 81% (68/83) of the provinces and 82% of the regions (14/17).

**Fig 5:**
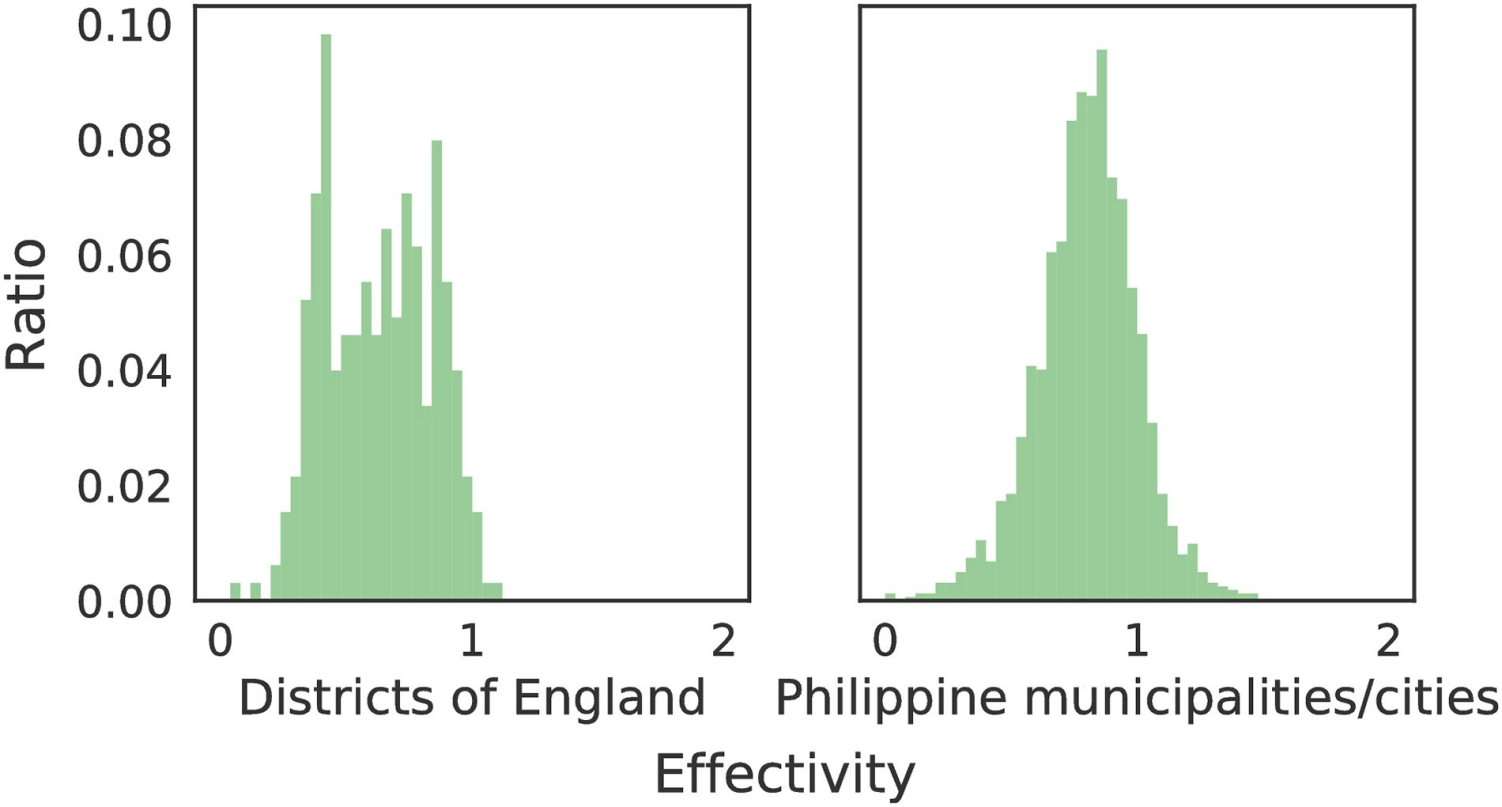
Distribution of the effectivity *E* of the UK school system and Philippine public school system at the district and municipality/city level, respectively.

To benchmark the effectivity metric, Fig 5 shows *E* for the UK primary school system and the Philippine public school system at the district and municipality/city level, respectively. The shapes of the distributions vary. Ninety-seven percent (97%, 319 out of 326) of districts in England have *E* < 1, suggesting that most English districts have fewer enrolled students than the school-aged residents in the same district. In the Philippines, 86% (1394 out of 1623) of the municipalities/cities have *E* < 1. Around 0.6% (2 out of 326) of districts in England have *E* < 0.2—there are only 2 students for every 10 school-aged residents in said districts. Roughly 0.7% (7 out of 1623) of municipalities/cities have *E* < 0.2. Areas with *E* > 1 suggest that our model assigned more students to the area than actual school-aged residents in the administrative unit.

It should be noted that the effectivity metric calculated from the England dataset may have been underestimated for some areas since there are schools with different starting age for school entry. The available data for student population contains the whole age range for all students. However, this should only shift the distribution and not affect its overall shape.

### Effects of imposing administrative boundary restrictions

The current public school system in the Philippines allows students to study in areas outside of their local residence. In this section, we determine the effect of imposing more restrictive boundaries. This may either be in the form of outright bans, stiffer non-resident fees, or limited support for non-residents.

It is important to consider multiple scales in our analysis, rather than just aggregating the results at a particular administrative level. Drawing geographic boundaries combined with aggregation can skew indicators, by intentionally or unintentionally masking out negative indicators. An example of which is gerrymandering, in which political boundaries are drawn to modify the outcome of a majority vote.

Stricter boundaries imply limiting the number of available schools for students residing near the boundaries. The reduction of school choices can change measured effectivity. An increase in *E* for a unit implies that schools which were originally catering to students across the administrative boundary now only cater to residents in that unit. Conversely, a decrease in *E* shows that imposing administrative boundaries has reduced the number of students from the unit that schools are accommodating. With the border restrictions, schools slots are redistributed for students residing within the administrative boundaries.

**Fig 6:**
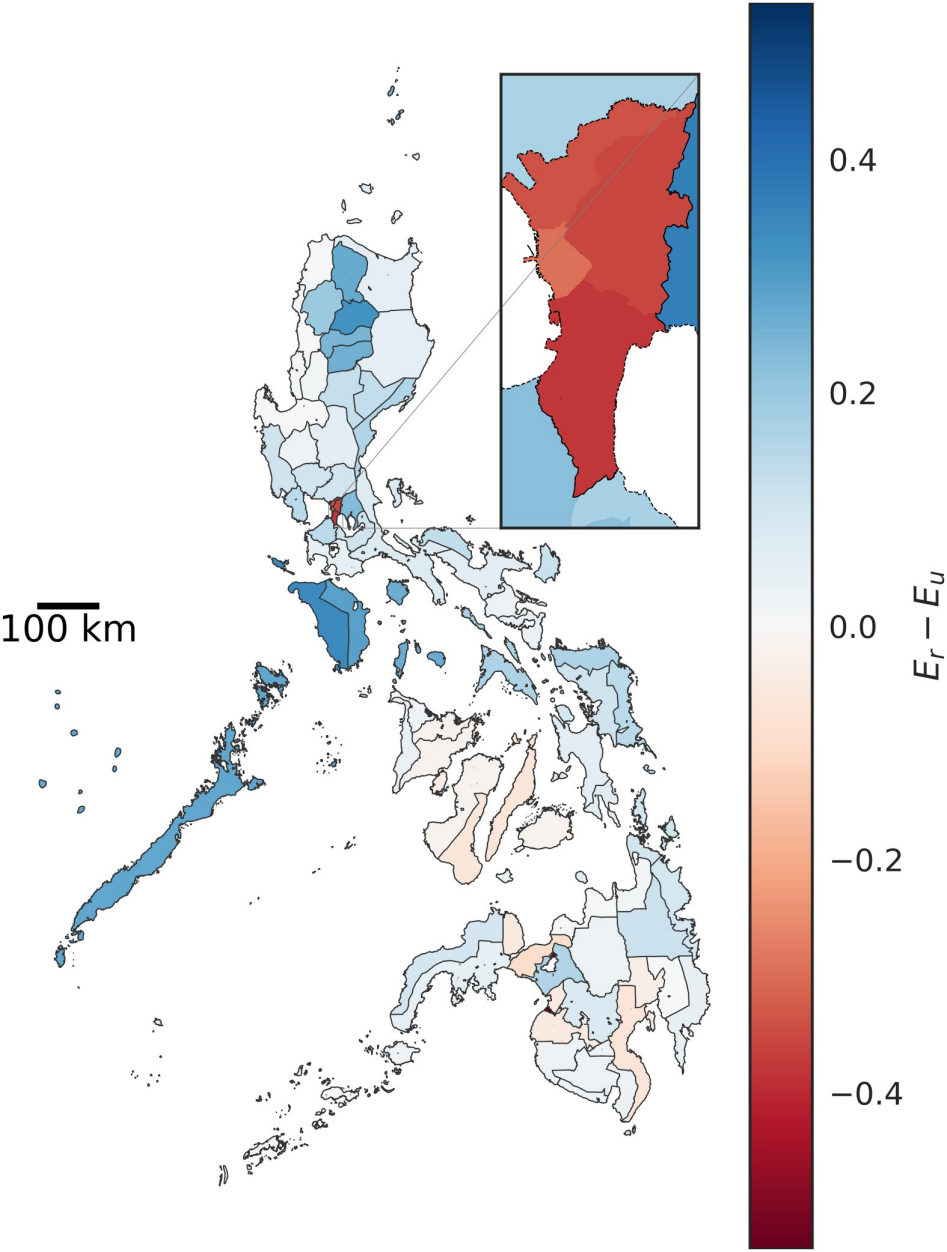
Difference in effectivity of provinces in the Philippines for the public elementary school system with imposed regional boundaries (*E*_*r*_) and without imposed boundaries (*E*_*u*_).

**Fig 7:**
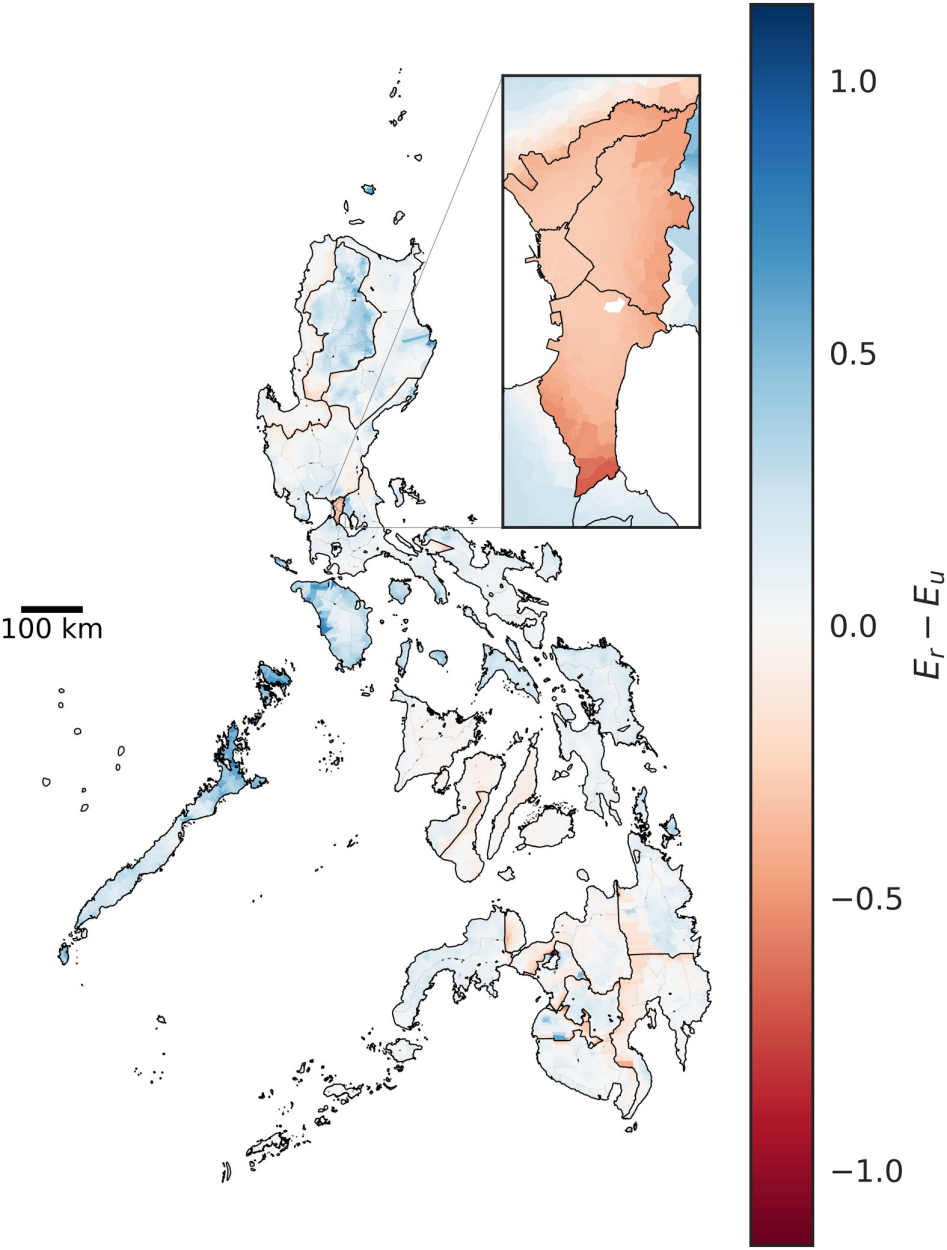
Difference in effectivity of barangays in the Philippines without aggregation for the public elementary school system with imposed regional boundaries (*E*_*r*_) and without imposed boundaries (*E*_*u*_).

From a modeling standpoint, it is interesting to see which administrative units would be most affected by imposing strict boundaries at different aggregation levels. Changes in effectivity are expected at the boundaries. However, depending on the level of aggregation of data, barangays that are negatively affected may be overlooked. As an example, *E* aggregated at the provincial level would suggest that regions in the northern Philippines generally benefit from the imposition of regional administrative boundaries (Fig 6). However, looking at the individual changes in *E* at the barangay level would show negatively affected barangays at the borders of the imposed boundary restrictions (Fig 7).

Another example is the case of the National Capital Region (NCR), which is subdivided into four districts. NCR comprises three districts and Manila, and accounts for 12.7% of the national population. With the imposition of regional administrative boundaries, *E* decreases for all provinces in NCR, while *E* increases for provinces in its neighboring regions. For the unbounded case, our model predicts that schools in NCR and its neighboring provinces are attended mostly by NCR residents. Imposing regional boundaries (Figs 6 and 7) cuts off the residents of NCR from attending schools in the neighboring provinces. This reduces the number of students, which lowers *E* in NCR. The barangays in the eastern and southern neighboring provinces (Fig 7) have an increased effectivity since their schools are now able to cater to their own residents rather than accommodating residents of NCR. However, barangays in the northern border of NCR (Fig 7) are negatively affected by the cut in NCR school access.

When stricter provincial boundaries are imposed, effectivity values are further reduced in Manila. This implies that Manila public schools alone cannot support its resident population. Boundary restrictions do not only negatively affect barangays at the borders; There are negative impacts particularly in highly dense areas. The source of such pressure is most likely rural-urban migration [33] and subsequent urban sprawl.

A significant caveat of this study is that the population projection is from the year 2000 school-age population projected to the year 2015 using the national growth rate. Thus, the effectivity metric, which is the ratio of slots in a given administrative unit to the school-aged children population in the same unit, could have a significant margin of error. That said, effectivity which is a better measure of resource strain, is significantly affected by having strict regional (Fig 4—*E*_*r*_) and provincial (Fig 4—*E*_*p*_) restrictions on school attendance.

## Conclusion

This work presents a data-centric way of quantifying public school accessibility, and enrollment pressure on the public school system, using a combination of a potential-based approach to spatial analysis, enrollment figures, and population data.

We can determine administrative units that have an excess or lack of school slots relative to that unit’s local population. Areas with low enrollment pressure can be developed further to help lessen the burden on other locations experiencing a higher enrollment pressure. Giving access to livelihoods in areas of low enrollment pressure would incentivize families to stay in these locations instead of moving into the crowded urban metropolis.

Using an effectivity metric, we can identify barangays that potentially do not have reasonable access to public school education. These would be barangays that are too far away from nearby schools, where in our model, a distance of 15 km would be considered sufficiently far away.

The effects of the aggregation of data at some administrative level, which is often how figures are reported to decision-makers, is also addressed. Aggregation of data results in a narrowing of the distribution of values, which can overshadow the extremes of metrics or indicators.

Our radial model of extent of schools did not consider the presence of bodies of water, nor significant changes in elevation. It also does not consider distances along a transportation network, which would be a more realistic method of handling distances. However, these factors can be accounted for by changing *r*_*c*_. A different *r*_*c*_ assignment per school can also be specified and assigned in proportion to the population ratio/group preference. For example, the population group of students who are good in math would assign a higher *r*_*c*_ value for schools that specialize in science and math.

Thus, accessibility values that are presented should be considered as upper bounds. Despite this limitation, geolocation information allows for multi-scale spatial modeling that readily yields insights on disparities that directly affect school enrollment. Our modeling scheme can be applied prospectively in the process of selecting future school locations. In addition, metrics other than school enrollment can be used as weights in our model.
